# Impact of late follicular luteinizing hormone levels on live birth rate after fresh embryo transfer: a retrospective cohort study in GNRH antagonist cycles

**DOI:** 10.3389/fendo.2025.1615525

**Published:** 2025-11-07

**Authors:** Nada Jelassi, Appoline Zimmermann, Cindy Faust, Laura Miquel, Christophe Buffat, Jeanne Perrin, Blandine Courbiere

**Affiliations:** 1 Department of Gynecology Obstetric and Reproductive Medicine, AP-HM La Conception University Hospital, Marseille, France; 2 Public Health Department, AP-HM Aix Marseille University, Marseille, France; 3 Biochemistry Department, Hôpital de la Conception, AP-HM, Marseille, France; 4 IMBE, Aix Marseille Univ, Avignon Univ, CNRS, IRD, Marseille, France

**Keywords:** IVF, GnRH antagonist, embryo implantation, live birth rate, luteinizing hormone (LH), fresh embryo transfer

## Abstract

**Objective:**

To investigate the impact of LH levels in the late follicular phase during the GnRH antagonist protocol on embryo implantation and IVF live birth rate (LBR) after fresh embryo transfer (ET).

**Design:**

Retrospective cohort study.

**Subjects:**

Women who underwent controlled ovarian stimulation (COS) with a GnRH antagonist protocol at a Reproductive Medicine Center in a University Teaching Hospital between January 2020 and December 2022.

**Exposure:**

Monocentric study involving 544 IVF cycles with the GnRH antagonist protocol. Four groups were stratified based on preovulatory LH levels: Q1: LH < 25th percentile, Q2: LH 25-50th percentile, Q3: LH 50-75th percentile, and Q4: LH > 75th percentile.

**Main outcome measures:**

The primary outcome was the live birth rate after fresh embryo transfer. Secondary outcomes included embryo implantation, clinical pregnancy, and early pregnancy loss rates.

**Results:**

During the late follicular phase, estradiol levels were significantly correlated with preovulatory LH levels (P = 0.03). The number of retrieved oocytes, 2PNs (two pronuclei), and usable embryos were similar across the groups. No significant differences were observed between the groups regarding implantation rate, clinical pregnancy rate, early pregnancy loss, and LBR (P > 0.05).

**Conclusion:**

In the antagonist protocol, pre-ovulatory LH levels had no impact on embryo implantation or IVF outcomes. A low LH level in the late follicular phase is not an indication for embryo freeze-all.

## Introduction

The GnRH antagonist protocol is recommended for IVF ovulation stimulation due to its comparable efficacy and higher safety compared to GnRH agonist protocols (1). GnRH antagonists bind to specific receptors on the pituitary gland, inhibiting endogenous luteinizing hormone (LH). During ovarian stimulation, GnRH antagonists prevent an early rise in LH, thereby reducing the cycle cancellation rate.

LH, secreted by the pituitary gland, is essential for normal follicular development and oocyte maturation in the menstrual cycle (2). Exogenous LH supplementation is necessary for folliculogenesis in cases of hypogonadotropic hypogonadism (3–5). However, the role of LH in embryo implantation remains controversial, particularly in women undergoing GnRH antagonist protocols (6–8). Indeed, GnRH antagonists induce pronounced suppression of LH during ovarian stimulation (9). Some studies suggest that profound suppression of LH during the follicular phase may negatively affect IVF outcomes with fresh embryo transfer (10–12). However, there is no consensus on the LH threshold that would be deleterious for embryo implantation. Zhou et al. suggested canceling fresh embryo transfers in the < 25th percentile group of LH levels (<1.62 mIU/mL in normal responders, <2.25 mIU/mL in PCOS women, and 2.14 mIU/mL in poor responders) (12). Conversely, other studies have not found such negative impacts of LH levels during the follicular phase (6, 13, 14). The impact of LH levels on endometrial receptivity could be either direct, through LH’s effect on the endometrium, or indirect, through estradiol or progesterone levels at the end of the follicular phase. Another hypothesis would be the direct impact of Antagonists on IVF results through action on the endometrium by immunomodulation.

This study aimed to evaluate the impact of LH levels at the end of the follicular phase on embryo implantation and live birth rate.

## Materials and methods

### Study design and patients

This retrospective study was conducted in a single IVF Unit at a University Teaching Hospital. We included all women aged 18–43 years undergoing a fresh embryo transfer using the GnRH antagonist protocol between January 2020 and December 2022. Exclusion criteria included hypothalamic or pituitary amenorrhea, indication for freeze-all (OHSS, increased progesterone, etc.), and missing core data. IVF cycles were stratified into four groups based on preovulatory LH levels.

### Study protocol

All women underwent a COS cycle using a fixed GnRH antagonist protocol. Women were pre-treated with estradiol (oral or transdermal patch) starting on the 25th day of the cycle preceding the stimulation cycle, lasting 5–10 days. Recombinant follicle-stimulating hormone (r-FSH) (without and with LH activity like Menopur) was administered on day 2 or 3 of the menstrual cycle, with the starting dose adapted to age, BMI, AMH concentration, and antral follicle count. The GnRH antagonist was started on day 5 of ovarian stimulation. From day 7 of stimulation, cycles were monitored by transvaginal ultrasonography and measurement of LH, progesterone, and estradiol. Recombinant-LH was not used in the cycles analyzed. When at least three follicles measured more than 17 mm, recombinant human chorionic gonadotropin (hCG) was administered at a dose of 250 micrograms to induce oocyte maturation. For poor responders, dual triggering was used. Oocyte retrieval was performed 36 hours after hCG administration. Fertilization was performed with conventional IVF or ICSI using fresh or thawed sperm.

Serum LH was measured at the last monitoring visit before hCG triggering, (on the day of triggering (J0), the day before (J–1), or two days before (J–2), depending on the individual monitoring schedule.

### Hormone assay

Serum LH levels were measured using the Atellica IM LH test (SIEMENS Healthineers). The intra- and inter-assay coefficients of variation were 2.7% and 3.7%, respectively.

### Fresh embryo transfer and luteal phase support

Fresh embryo transfers were primarily performed on day 2 or 3, with some on day 5. The embryos transferred were among the usable embryos (defined as diploid embryos used for fresh ET or freezing). The number of embryos transferred (one or two) was determined by embryo grading (15), the woman’s medical history, and the couple’s choice. According to Griesinger et al., oral dydrogesterone 30 mg/day was prescribed for LPS after fresh ET starting on the day of oocyte pick-up (16).

### Outcome measures

The primary outcome was the live birth rate after fresh ET. Secondary outcomes included clinical pregnancy, defined as the detection of fetal heart activity by transvaginal ultrasonography 5 weeks after embryo transfer; implantation rate, calculated as the number of intrauterine gestational sacs observed by transvaginal ultrasonography divided by the total number of transferred embryos; and early pregnancy loss, defined as the spontaneous loss of clinical pregnancy before 12 weeks of gestation.

### Statistical analysis

We determined that including 236 cycles would provide 80% power at a two-tailed alpha level of 0.05 to detect a significant difference in LBR (absolute difference was 12.1%) between the group with the lowest preovulatory LH levels and the group with the highest preovulatory LH levels, with an OR of 2.56. First, a descriptive analysis of the entire sample was performed. Categorical variables were presented as proportions and numbers, while quantitative variables were presented as mean ± standard deviation or median and quartiles. For each variable, the proportion of missing data was specified. A comparative analysis was conducted according to quartiles of LH levels at the last blood test. Qualitative variables were compared using Chi-square or Fisher’s exact tests according to application conditions, and quantitative variables were compared using a one-way ANOVA test followed by Bonferroni *post-hoc* tests for multiple comparisons. Pregnancy outcomes were also compared using the same procedure. The results of the analysis are presented as bar charts with their 95% confidence intervals (95% CI). The significance threshold was set at 5%. All analyses were carried out using IBM SPSS^®^ Statistics version 20 software.

### Ethical statement

The study protocol was approved by the local ethics committee (PADS23-71).

## Results

A total of 552 IVF cycles with fresh ET were included in the study. Preovulatory LH level data were missing for 8 cycles, which were excluded from the analysis. Consequently, 544 IVF cycles with fresh ET were analyzed. The women included had a mean age of 35.3 ± 4.6 years, a mean BMI of 25.8 ± 5.2 kg/m², and 17% were smokers (n = 94).

**Figure 1:**
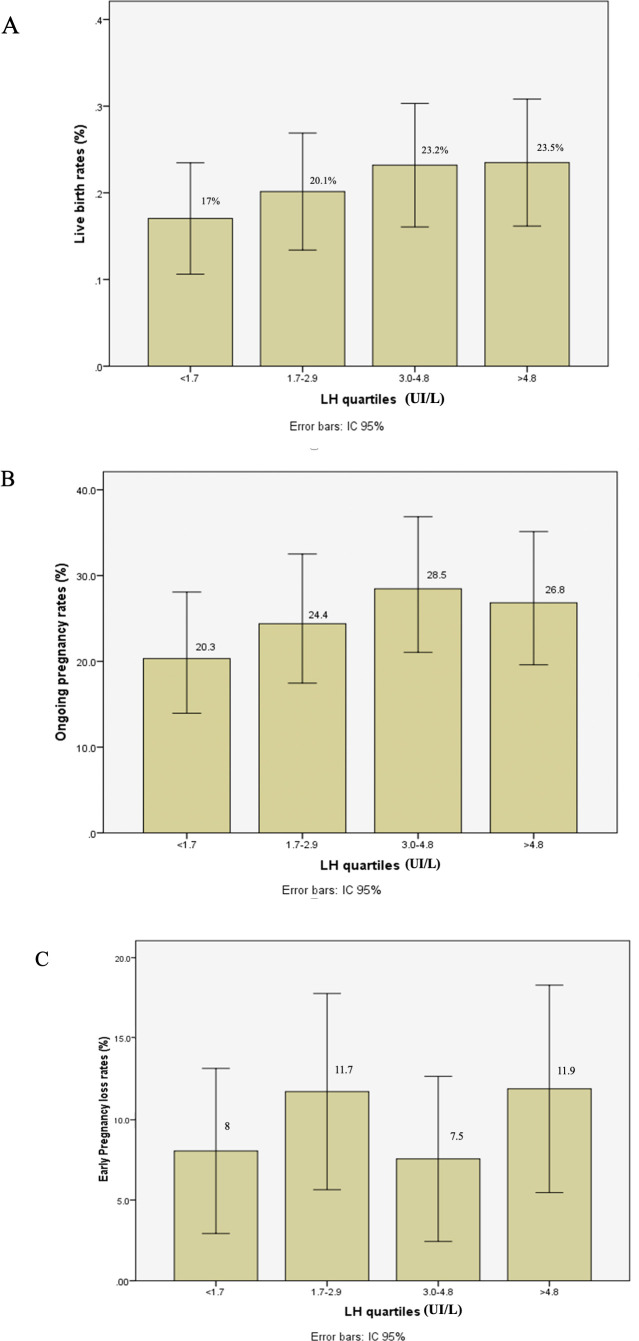
Live birth rate **(A)**, ongoing pregnancy rate **(B)**, and early pregnancy loss rate **(C)** after fresh embryo transfer according to LH levels in late follicular phase during GnRH antagonist protocol.

The IVF cycles were categorized into four groups according to the interquartile range of preovulatory LH levels: Q1 = LH < 1.7 UI/L; Q2 = LH 1.7-2.9 UI/L; Q3 = LH 2.9-4.8 UI/L; Q4 = LH > 4.8 UI/L (**Figure 1**). The characteristics of the women in each of the four groups are detailed in **Table 1**. No significant differences were found among the groups regarding age, BMI, tobacco use, duration of infertility, type of infertility (primary or secondary), and cause of infertility. The causes of male and female infertility were comparable across all groups. The percentage of women with diminished ovarian reserve was 31.3% (n = 31) in Q1, 43% (n = 40) in Q2, 40% (n = 40) in Q3, and 37.4% (n = 34) in Q4 (p= 0,5). The proportion of poor responders was comparable across the four LH quartiles (31–43%), with no statistically significant difference between groups. The percentages of tubal anomalies were 16.2% (n = 16) in Q1, 5.4% (n = 5) in Q2, 12% (n = 12) in Q3, and 6.6% (n = 6) in Q4, with no statistical significance.

**Table 1:** Characteristics of women underwent GnRH antagonist protocol based on late follicular phase LH levels.

	LH< 1.7 (135)	LH [1.7-2.9] (139)	LH [2.9-4.8] (138)	LH > 4.8 (132)	P value
Age(y)*	34.8 ± 4.8	35 ± 4.5	35.4 ± 4.6	36.1 ± 4.4	0.12
BMI (kg/m2)*	25.7 ± 5.5	26 ± 5.1	25.4 ± 4.8	26.2 ± 6	0.62
Tabacco use**	19.3	18	14.6	17.4	0.77
Duration of infertility (m)*	51.3 ± 34	60.9 ± 37.6	58.2 ± 32.1	60.7 ± 38.9	0.09
Type of infertility**					0.91
Primary	63	59	61.6	59.8	
Secondary	37	41	38.4	40.2	
Cause of infertility **					0.8
Female	36.9	40.3	40.2	35.8	
Male	23.1	27.9	21.3	26	
Mixed	24.6	20.9	26.8	24.4	
Unexplained	13.8	9.3	11	10.6	
Other	1.5	1.6	0.8	3.3	
Basal LH(UI/L)*	5.4 ± 3.2	5.5 ± 3.3	5.6 ± 2.3	6.4 ± 3	0.03
Basal FSH(UI/L)*	6.6	7.4	7.2	7.4	0.01
AMH (ng/ml)*	2.7	2.3	2.4	2.4	0.3

Unit of measurement for LH was UI/L.

*Data are expressed as mean ± SD, **Data are presented as %, (m) = Month.

The mean level of basal LH before stimulation was significantly higher in Q4 compared to Q. Mean AMH levels were 2.7 ± 2.3 ng/mL in Q1, 2.3 ± 2 ng/mL in Q2, 2.4 ± 1.8 ng/mL in Q3, and 2.4 ± 1.7 ng/mL in Q4 (P = 0.3). Significant differences were observed between Q1 and Q4 regarding preovulatory estradiol levels (1837.5 ± 1025.6 pg/mL vs. 2159.9 ± 827.2 pg/mL; P = 0.025) and between Q2 and Q4 in terms of the duration of stimulation (11.1 ± 1.7 days vs. 10.5 ± 1.7 days; P = 0.026) and the total dose of gonadotropin (3303.8 ± 1310.3 UI vs. 2875.4 ± 1291.9 UI; P = 0.04). Other COS parameters, such as preovulatory progesterone levels, endometrial thickness, type of gonadotropin used (with or without LH activity), and the method of ART, were comparable across all groups (**Table 2**).

**Table 2:** GnRH antagonist cycle characteristics based on late follicular phase LH levels.

	LH< 1.7 (135)	LH [1.7-2.9] (139)	LH [2.9-4.8] (138)	LH > 4.8 (132)	P value
Duration of stimulation (Day)*	11.2 ± 1,6	11.1 ± 1.7	11 ± 1.6	10.5 ± 1.7	0.06
Total dose of gonadotrophin*	2972.3 ± 1270.6	3303.8 ± 1310.3	3196.4 ± 1306	2875.4 ± 1291.5	0.03
Last E2 during stimulation*	1837.5 ± 1025.6	2011.9 ± 941.9	2072.2 ± 853.4	2159.9 ± 827.2	0.03
Last P4 during stimulation	0.7 ± 0.5	0.7 ± 0.4	0.8 ± 0.4	0.8 ± 0.4	0.2
Gonadotrophin used**					0.16
Without LH activity	63.7	68.3	57.2	68.9	
With LH activity	36.3	31.6	42.8	31.1	
Last measure of endometrium (mm)*	10.6 ± 2.6	10.8 ± 2.6	11 ± 2.6	10.5 ± 2.3	0.3
Method of fertilization**					0.15
IVF	62.2	57.6	55.8	51.5	
ICSI	37.8	42.4	44.2	48.5	
No.oocytes retrieved*	10.7 ± 5.4	9.4 ± 5.5	9.7 ± 4.7	9.6 ± 5.4	0.2
No. of usable embryos*	2.4 ± 1.2	2.2 ± 1.2	2.3 ± 1.3	2.3 ± 1.3	0.7
Embryo transferred**					0.7
J5 or J6	11.9	14.4	13	9.8	
J2 or J3	88.1	85.6	87	90.2	

Unit of measurement for LH was UI/L.

*Data are expressed as mean ± SD, **Data are presented as %,

The number of oocytes retrieved, and the number of usable embryos were similar across all groups. IVF outcomes are presented in **Table 3** and **Figure 1**. After fresh embryo transfer, there were no significant differences among the four groups regarding implantation rate, clinical pregnancy rate, early pregnancy loss, and live birth rate.

**Table 3:** Outcomes of GnRH antagonist cycles after fresh embryo transfer based on late follicular phase LH levels.

Outcomes	LH< 1.7 (135)	LH [1.7-2.9] (139)	LH [2.9-4.8] (138)	LH > 4.8 (132)	P value
Implantation rate**	24.8	25.6	25.4	24.3	0.69
Clinical pregnancy rate**	18.5	21.6	25.4	25	0.5
Early pregnancy loss°	8(112)	11.7(111)	7.5(106)	11.9(101)	0.58
Live Birth rate**	17	20.1	23.2	23.5	0.53

Unit of measurement for LH was UI/L.

**Data are presented as %, °Data are presented as % and (n-total).

## Discussion

In our study of 544 GnRH antagonist cycles, the late follicular LH level did not influence the implantation rate or LBR after fresh ET. The increase in preovulatory estradiol levels was associated with the preovulatory LH level. Our outcome supports the conclusion from other literature that shows pre-ovulatory estradiol levels do not make a difference on IVF outcomes (17–19). Despite the correlation between LH and estradiol levels, Huang et al. found no impact of peri-implantation estradiol levels on IVF pregnancy outcomes (18).

Taken together, these findings suggest that although LH and estradiol levels are associated, their fluctuations do not appear to significantly affect clinical outcomes in IVF.

Some studies have suggested that low LH levels at the end of the follicular phase might negatively affect the endometrium and IVF outcomes with GnRH antagonists and fresh ET (10–12, 20). If confirmed, this hypothesis could make low LH levels in the late follicular phase an indication for embryo freeze-all.

Shoham et al. proposed an “LH window” necessary for follicular development, emphasizing the need for a minimum circulating endogenous LH level for adequate ovarian steroidogenesis (21). Adding “LH activity” during COS is recommended for women with a poor response to stimulation despite normal ovarian reserve. Conforti et al. found that women with a hypo-response to ovarian stimulation supplemented with recombinant LH (r-LH) had significantly higher clinical pregnancy rates, implantation rates, and number of oocytes retrieved than those who underwent r-FSH alone (22).

However, our data, together with most recent findings, suggest that low LH levels in antagonist cycles do not necessarily compromise oocyte quality or endometrial receptivity, and therefore should not automatically trigger a freeze-all approach.

This body of evidence highlights that while low LH levels may raise concerns, the clinical need for intervention such as embryo freeze-all remains debatable and context-dependent.

Regarding studies that observed an impact of LH levels on embryo implantation, the debate focuses on the LH threshold with conflicting results (10–12, 20). Few studies have investigated the effect of LH levels on LBR after fresh embryo transfer with the GnRH antagonist protocol. Our findings are consistent with those of Merviel et al., who found no significant difference in clinical outcomes between different LH levels on the day of hCG administration, with an arbitrary threshold of 0.5 IU/L (6). Similarly, Griesinger et al. reported no association between LH concentrations on day 8 of stimulation and ongoing pregnancy rates (13). Bosch et al. as well as Doody et al., also observed no differences in clinical pregnancy rates (23, 24).

In a study of 426 cycles, Eftekhar et al. reported no relationship between LH levels during GnRH antagonist cycles and pregnancy outcomes, though they did observe a higher implantation rate in the LH 2.60–4.60 IU/L group compared to the LH <1.49 IU/L group, but no significant difference between the LH <1.49 IU/L group and the LH >4.6 IU/L group (14).

These consistent findings reinforce our conclusion that there is no clear or universally applicable LH threshold predictive of live birth in antagonist cycles.

Conversely, Luo et al. showed that LH levels <4 IU/L significantly reduced LBR (38% vs 51.5%; P<0.05) and increased early pregnancy loss rate after fresh embryo transfer, without affecting the implantation rate. They included 1480 women undergoing COS with GnRH antagonist, who were arbitrarily divided into “low” and “high” LH groups with a cutoff of 4 IU/L (20). In the study of Benmachiche et al., 322 infertile women were included and underwent IVF with GnRH antagonist protocol and fresh ET. Above an LH threshold of 1.6 IU/L, early pregnancy loss rate decreased and the ongoing pregnancy rate and LBR increased. The absolute difference between the highest LH group (LH >1.6 IU/L) and the lowest LH group (LH<0.6 IU/L) was 13.4%, 12.1%, and 12% in ongoing pregnancy rate, LBR, and early pregnancy loss rate, respectively (P<0.05) (11). These discrepancies may be explained by differences in study design and patient populations. Luo et al. included a larger cohort with a higher proportion of PCOS women and used an arbitrary LH cutoff of 4 IU/L, whereas Benmachiche et al. applied a lower threshold of 1.6 IU/L. In addition, the LH assays and the timing and dosing of antagonist administration varied between studies, which may also account for heterogeneity in the reported outcomes.

These discrepancies highlight that the thresholds proposed in the literature can be considered specific to the study population rather than universal biological thresholds.

In a retrospective study, Chen et al. found that LH ≤0.8 mIU/ml during COS with GnRH antagonist was associated with a significantly higher early pregnancy loss rate (31.1%, n=19 vs 16.6%, n=36; P = 0.012) but no significant differences in LBR or implantation rate (10). There is variability in LH levels during COS with the antagonist protocol, with differences described between the first day of the cycle and after introduction of GnRH antagonists (25). For long-agonist protocol, Lahoud et al. observed that a drop in LH levels of >50% from early to mid-follicular phase resulted in a significantly lower live birth rate per cycle (22.2% vs 15.8%; P<0.05) (26). Despite the use of the same dose of GnRH antagonists, women do not have the same pituitary response. Kol et al. defined “normal” responders as those with LH levels greater than 50% of the pre-injection level 24 hours after the first GnRH antagonist injection and “over-suppressed” if less than 50%. About a quarter of women were “over-suppressed” in their study, though they did not investigate the impact on LBR (25). This variability in LH levels may be due to interindividual differences in pituitary response to GnRH antagonists, potentially explained by polymorphisms in the GnRH receptor (GnRHR). In a study of 269 women, Weng et al. found that genetic variants in the GnRHR gene could modulate LH release and affect ovarian stimulation outcomes (27).

LH receptor polymorphisms appear to have no impact on clinical outcomes or IVF LBR. In a study of 1183 women in whom genotyping of the FSH receptor and LH receptor polymorphisms was performed, Pirtea et al. found no association between gonadotropin receptor polymorphisms and LBR or implantation rate (28).

Altogether, these findings suggest that variability in LH response may be influenced by genetic and pharmacological factors, but these do not consistently translate into differences in live birth outcomes.

To understand the mechanism of action of GnRH antagonists, Murase et al. reported that prolonged GnRH antagonist treatment inhibits LH beta subunit mRNA and GnRH-R mRNA expression, like GnRH agonists (29). In a review, Maggi et al. reported that the GnRH/GnRH-R system is also expressed in female reproductive tissues, in addition to the pituitary gland. The expression of this system in the human endometrium supports its physiological role in the processes of embryo implantation (30). A comparative transcriptomic analysis study of the endometrium in 9 women with adenomyosis before and after GnRH agonist treatment suggested that GnRH agonists significantly alter immune system-associated signal transduction in the endometrium, a hypothesis that remains unexplored for GnRH antagonists (31).

These mechanistic data highlight the need for future research investigating how GnRH antagonists might influence endometrial gene expression and immune modulation, potentially mediating subtle effects on implantation.

Despite the retrospective and monocentric nature of our study, as well as the heterogeneity of the population, our sample was sufficiently large, even though we had few women with LH levels below 0.6 IU/L.

We acknowledge as a major limitation the retrospective cohort design, which may introduce selection bias and limits causal inference.

Overall, our study adds value by providing an analysis of a large sample of late follicular phase LH levels, with a focus on the results of fresh embryo transfers, which helps to guide clinical decision-making. Our findings suggest that low late follicular LH levels alone should not be used as a criterion to systematically opt for a freeze-all strategy. Instead, clinicians can continue to perform fresh transfers even in cases of low LH, provided that no other clinical or biological factors indicate otherwise. This can help avoid unnecessary delays in treatment and reduce patient burden.

However, as this is a descriptive study based on statistical associations, it cannot establish a causal relationship between late follicular LH levels and IVF outcomes. Therefore, our results should be interpreted with caution. Further prospective multicenter and mechanistic studies are needed to confirm these findings and to better understand the pathways underlying LH variability and its potential impact on endometrial receptivity.

In conclusion, our study indicates that preovulatory LH levels during cycles with GnRH antagonists are not correlated with IVF outcomes. Therefore, monitoring LH levels during antagonist protocols may be of limited use in deciding between fresh transfer or freeze all, and a low LH level at the end of the follicular phase does not necessarily require freezing all.

## Data Availability

The original contributions presented in the study are included in the article/supplementary material. Further inquiries can be directed to the corresponding author.
